# Bile Acid Regulates Mononuclear Phagocytes and T Helper 17 Cells to Control *Candida albicans* in the Intestine

**DOI:** 10.3390/jof8060610

**Published:** 2022-06-07

**Authors:** Abhishek Datta, Juan F. Hernandez-Franco, Sungtae Park, Matthew R. Olson, Harm HogenEsch, Shankar Thangamani

**Affiliations:** 1 Department of Comparative Pathobiology, College of Veterinary Medicine, Purdue University, West Lafayette, IN 47906, USA; datta42@purdue.edu (A.D.); jfhernan@purdue.edu (J.F.H.-F.); hogenesc@purdue.edu (H.H.); 2 Department of Biological Sciences, Purdue University, West Lafayette, IN 47906, USA; park885@purdue.edu (S.P.); olson126@purdue.edu (M.R.O.); 3 Purdue Institute for Immunology, Inflammation and Infectious Diseases (PI4D), West Lafayette, IN 47906, USA

**Keywords:** bile acids, innate and adaptive immunity, fungal colonization

## Abstract

Invasive *Candida albicans* (**CA**) infections often arise from the intestine and cause life-threatening infections in immunocompromised individuals. The role of gut commensal microbiota, metabolites, and host factors in the regulation of CA colonization in the intestine is poorly understood. Previous findings from our lab indicate that taurocholic acid (**TCA**), a major bile acid present in the intestine, promotes CA colonization and dissemination. Here, we report that oral administration of TCA to CA-infected mice significantly decreased the number of mononuclear phagocytes and CD4+ IL17A+ T helper 17 cells that play a critical role in controlling CA in the intestine. Collectively, our results indicate that TCA modulates mucosal innate and adaptive immune responses to promote CA colonization in the intestine.

## 1. Introduction

*Candida albicans* (**CA**) is an opportunistic fungus that frequently inhabits the gastrointestinal (GI) tract and can cause an invasive fungal infection [1,2,3,4,5]. Dysregulation in host immunity and (or) gut microbiota dysbiosis are the major predisposing factors that contribute to increased CA colonization and (or) dissemination from the intestine [6,7,8,9,10]. Therefore, understanding the factors that regulate CA colonization and dissemination will lead to the identification of novel approaches to prevent and treat life-threatening fungal infections in humans [11,12,13,14]. To address this gap in the knowledge, we utilize a combination of targeted metabolomics, 16S ribosomal RNA amplicon gene sequencing, and in-vivo mouse models of CA infection to identify the metabolites, microbiome, and host factors regulating CA colonization in the intestine [13,14].

Our previous findings indicate that taurocholic acid (**TCA**), a major bile acid present in both humans and mice, controls the balance between commensalism and invasive CA infection originating from the gut [14]. Oral administration of TCA through drinking water is sufficient to induce CA colonization and dissemination from the intestine, even in the absence of antibiotic and (or) immunosuppressive treatment [14]. Furthermore, TCA alters the relative abundance of certain commensal bacterial members and the expression of antifungal peptides in the colon of mice that had increased fungal colonization and dissemination [14]. Since innate (neutrophils and macrophages) and adaptive immune cells (T helper 1 and 17 cells) contribute to antifungal defense in the intestine [5,8,15,16], we aim to understand if TCA dysregulates mucosal immune response to promote CA colonization in the intestine. Our results suggest that TCA treatment significantly decreased the mononuclear phagocytes and T helper 17 cells that play an important role in antifungal defense against CA in the intestine.

## 2. Materials and Methods

**Strains and Reagents.***Candida albicans* SC5314 was provided by Dr. Andrew Koh from the University of Texas Southwestern Medical Center. The following reagents and chemicals were purchased from the indicated vendors below. Yeast Peptone Dextrose (YPD) (242810, BD Difco, Franklin Lakes, NJ, USA), agar (BP1423-500, Fisher Bioreagents, Pittsburg, PA, USA, RPMI-1640 (SH30027.02, Hyclone-Cytiva Marlborough, MA, USA), fetal bovine serum (FBS-500-H, CPS Serum, Parkville, MO, USA), taurocholic acid (16215, Cayman Chemical, Ann Arbor, MI, USA), HEPES (BP310-500, Fisher Bioreagents, Pittsburg, PA, USA), L-glutamine (G3126, Sigma-Aldrich, St. Louis, MO, USA), penicillin-streptomycin (P4333-100ML, Sigma-Aldrich, St. Louis, MO, USA), gentamycin (15710-072 Life Technologies, Carlsbad, CA, USA), D-glucose (D16-500, Fisher Bioreagents, Pittsburg, PA, USA), sodium bicarbonate (S233-500, Fisher Chemical, Pittsburg, PA, USA), 10× Hanks Balanced Salt Solution (HBSS) without calcium chloride, magnesium sulfate and sodium bicarbonate (20-021-CV, Corning, Corning, NY, USA), 0.5 M EDTA (15575-020, Life Technologies, Carlsbad, CA, USA), collagenase Type 1 (17100-017, Life Technologies, Carlsbad, CA, USA), MgCl_2_ hexahydrate (M2670 Sigma-Aldrich, St. Louis, MO, USA), CaCl_2_ hexahydrate (21108, Sigma-Aldrich, St. Louis, MO, USA), percoll (GE17-0891-01, Millipore–Sigma, Burlington, MA, USA), 10× phosphate buffer saline (BP-3994, Fisher Bioreagents, Pittsburg, PA, USA), falcon 70 μm cell strainer (08-771-2, Fisher Scientific, Waltham, MA, USA). U-bottom 96 well Plate (229190) from Cell Treat (Pepperell, MA, USA) was used. Anti-mouse antibodies and staining reagents were purchased from Biolegend, San Diego, CA, USA; antibodies (CD16/32, CD3ε, CD4, CD8a, Ly-6G, CD11b, F4/80, CX3CR1, CD45/B220, IFNγ, IL17A, IL5), cell staining and fixation buffer, cell activation cocktail without brefeldin A (500×), monensin solution (1000×) and intracellular staining perm wash buffer (10×).

**Mouse Infection Studies.** Male and female wild-type C57BL/6 mice between 7 and 12 weeks old co-housed in our animal facility were infected with *C. albicans* SC5314 via oral gavage with a dose of ~1 × 10^7^ CFU/mouse. After infection, the untreated groups received sterile drinking water and the TCA group received water containing 1% TCA in the drinking water as previously described [14]. After seven days of infection and treatment, the fungal load in feces was determined using antibiotic-containing YPD plates as described before [14]. On day eight, the mice were euthanized to determine the immune cell population in the intestine.

**Isolation of Cells from Intestinal Lamina Propria (LP).** Mononuclear cells from small and large intestinal LP were isolated as described elsewhere [17,18]. The intestines removed from the mice were kept in cold harvest media (RPMI 1640, 5%FBS, 5 mM HEPES, 2 mM L-glutamine, 20 U/mL penicillin, 20 µg/mL streptomycin, 0.05 µg/mL gentamycin, 4 mM NaHCO_3_, 10 mM D-glucose). Mesenteric fat and fecal material in the intestine were removed, and the intestine was washed with 1× Phosphate Buffer Saline. The intestinal mucus was removed by gently skimming the luminal wall of the intestine with curved forceps on tissue paper moistened with harvest media and were cut into 1–2 cm long pieces. Intestinal pieces were washed 3 times with EDTA solution (1× HBSS, 1.3 mM EDTA, 5 mM HEPES, 2 mM L-glutamine, 20 U/mL penicillin, 20 µg/mL streptomycin, 0.05 µg/mL gentamycin, 4 mM NaHCO_3_) containing dithiothreitol (1 mM). Washed intestinal pieces were then digested with collagenase solution (RPMI 1640, 10% FBS, 5 mM HEPES, 2 mM L-glutamine, 20 U/mL penicillin, 20 µg/mL streptomycin, 0.05 µg/mL Gentamycin, 1 mM MgCl_2_, 1 mM CaCl_2_, Type 1 collagenase 100 U/mL). The resulting digest was strained through a 70-micron cell strainer and centrifuged to collect the cells. The cell pellet was resuspended in 40% Percoll (Millipore Sigma, St. Louis, MO, USA) and centrifuged to remove all the cell debris. The final cell pellet was resuspended in 1 ml of complete RPMI media (RPMI 1640, 10% FBS) and the cells were counted using Trypan Blue. Small intestinal tissues of two mice from which we failed to isolate mononuclear cells were not included. Approximately one million cells were used for labeling.

**Cell Labeling and Flow Cytometry.** Cells were labeled using U-bottom 96 well plate (Cell Treat). For surface staining, cells were washed with cell staining buffer (420201, Biolegend) and Fc receptors were blocked with anti-mouse CD16/32 (Clone 93). The respective antibodies for surface markers, Ly-6G (PE/Cyanince7, 1A8), CD11b (PE, M1/70), F4/80 (Alexa Fluor 700, BM8), CX3CR1 (Alexa Fluor 488, SA011F11), CD3ε (FITC, 145-2C11), CD4 (APC, GK1.5) and B220 (PE, RA3-6B2), were used in a ratio of 1:100. After surface staining, cells were fixed using fixation buffer (420801, Biolegend). For intracellular cytokine staining, cells were stimulated for 4.5 h with cell activation cocktail (423301) and monensin (420701) at 37 °C at 5% CO_2,_ according to the manufacturer’s instructions. Then, cells were incubated with 1× intracellular staining perm wash buffer (421002) with antibodies; anti-mouse IFNγ (Alexa Fluor 700, XMG1.2), IL17A (PE/Dazzle 594, TC11-18H10.1) and IL5 (PE, TRFK5). Cells were washed and the flow cytometry data was acquired with a Attune NxT Flow Cytometer (Invitrogen, Carlsbad, CA, USA) and analyzed using FlowJo software (Eugene, OR, USA).

**Statistical analysis.** Statistical analyses were performed using GraphPad Prism 6.0 (Graph Pad Software, La Jolla, CA, USA). *p* values were calculated using Mann-Whitney test as indicated. *p* values of (* ≤0.05) (** ≤0.01) (*** ≤0.001) were considered as significant.

## 3. Results and Discussion

**TCA significantly decreased the mononuclear phagocytes in the intestine.** Macrophages and neutrophils are the major innate immune cells that play a critical role in host defense against CA in the intestine [8,9,19]. To identify if TCA dysregulates intestinal innate defense to induce CA colonization, we infected mice with CA and treated them with TCA-containing water or water only to determine the effect on the neutrophil and macrophage population in the intestinal lamina propria (LP). CA-infected mice that received 1% TCA in the drinking water had a significantly higher fungal load in feces, which was consistent with our previous findings (Supplementary Figure S1) [14]. Based on our previous findings, the onset of CA mortality in TCA-treated mice usually occurred eight days after infection and treatment [14]. Therefore, we examined the immune cell population in untreated control and TCA-treated groups on day 8.

**Figure 1:**
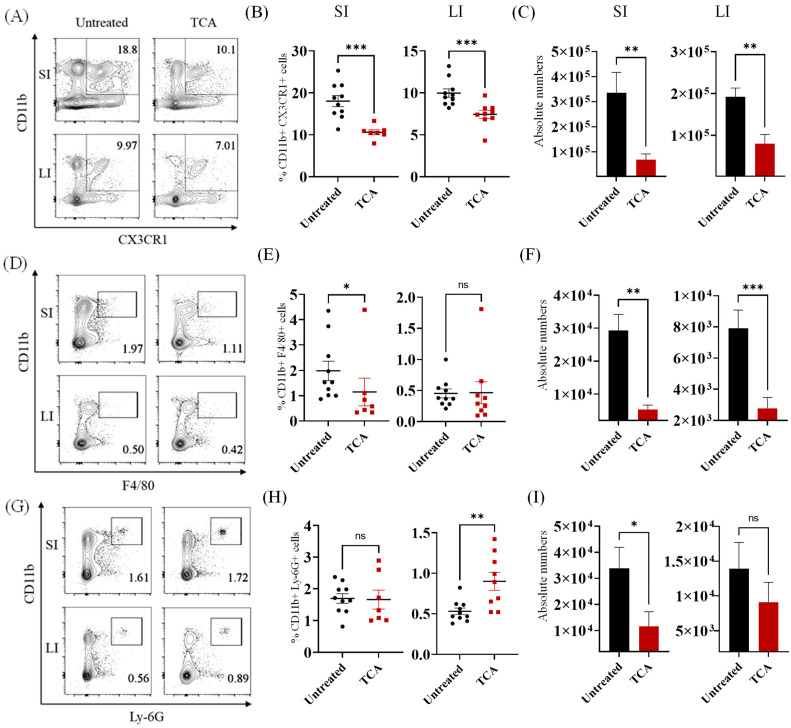
TCA significantly decreased the mononuclear phagocytes in the intestine. Groups of mice infected with ~1 × 10^7^ CFU of CA SC5314 via oral gavage. Control group received sterile drinking water and TCA group received drinking water containing 1% TCA. Eight days post-infection and treatment, mice were euthanized to isolated mononuclear cells from small and large intestine. Cells were labeled with indicated markers to determine macrophages and neutrophils population in the intestine. (**A**) Representative images; (**B**) percentage and (**C**) absolute number of CD11b+ CX3CR1+ phagocytes from small intestine (SI) and large intestine (LI) of untreated and TCA-treated mice were shown. (**D**) Representative images; (**E**) percentage and (**F**) absolute number of CD11b+ F4/80+ macrophages from SI and LI of untreated and TCA-treated mice were shown. (**G**) Representative images; (**H**) percentage and (**I**) absolute number of CD11b+ Ly6G+ neutrophils from SI and LI of untreated and TCA-treated mice were shown. Data shown are combined from 2 independent experiments for a total of 7 to 10 mice per group. Data represent mean ± SEM. The statistical significance of differences between groups was determined by the Mann-Whitney U test with * *p* ≤ 0.05, ** *p* ≤ 0.01, and *** *p* ≤ 0.001.

The percentage and absolute number of CD11b+ CX3CR1+ phagocytes were significantly decreased in the LP of small and large intestine of TCA-treated groups compared to untreated control groups (Figure 1A–C). This is consistent with the decreased relative expression of the *Cx3cr1* gene in the colon of TCA-treated mice compared to untreated mice infected with CA [14]. The percentage of CD11b+ F4/80+ macrophages was significantly decreased in the LP of the small but not in the large intestine of the TCA-treated groups. However, the absolute number of CD11b+ F4/80+ macrophages was decreased in both the small and large intestine of TCA groups (Figure 1D–F). Neutrophils showed a mixed trend. While the percentage of CD11b+Ly-6G+ neutrophils was significantly increased in the large intestine, the absolute number of neutrophils was significantly decreased in the small intestine of TCA treated groups (Figure 1G–I). Collectively, our findings indicate that oral TCA downregulates CD11b+ CX3CR1+ phagocytes and CD11b+ F4/80+ macrophages that play a critical role in antifungal defense to CA in the intestine [19,20].

Recent evidence indicates that antibiotic treatment impairs the recruitment of CX3CR1+ phagocytes in the intestine, resulting in bacterial translocation. Furthermore, the depletion of gut microbiota impairs the turnover of CX3CR1+ phagocytes in the colon [21,22]. Mice infected with CA and orally administered 1% TCA had a reduced relative abundance of several commensal bacteria, including *Turicibacter sanguinis, Lactobacillus johnsonii*, and *Clostridium celatum* [14]. Future studies will aim to elucidate whether TCA regulates CD11b+ CX3CR1+ phagocytes by modulating one or more of these microbiota members and will (i) expand the knowledge about the role of bile acids and commensal bacteria in the regulation of intestinal phagocytic cells and (ii) provide an in-depth understanding of bile-mediated regulation of CA colonization through intestinal phagocytes.

**Figure 2:**
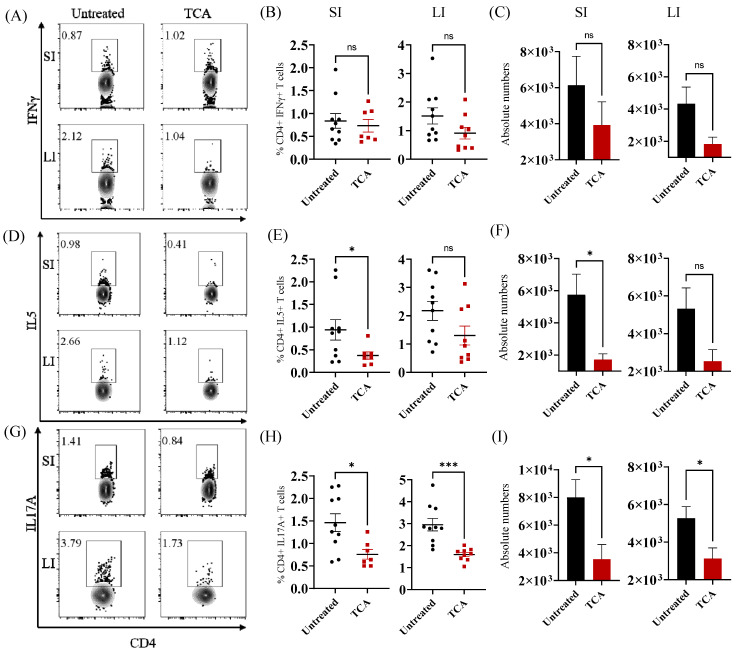
TCA significantly decreased Th2 and Th17 cells in the intestine. Groups of mice infected with ~1 × 10^7^ CFU CA SC5314 via oral gavage. Control group received sterile drinking water and TCA group received drinking water containing 1% TCA. Eight days post-infection and treatment, mice were euthanized to isolated mononuclear cells from small and large intestine. Cells were labeled with indicated markers to determine Th1, Th2 and Th17 population in the intestine of CA-infected mice. (**A**) Representative images; (**B**) percentage and (**C**) absolute number of CD4+ IFNγ+ T helper 1 cells from small intestine (SI) and large intestine (LI) of untreated and TCA-treated mice were shown. (**D**) Representative images; (**E**) percentage and (**F**) absolute number of CD4+ IL5+ T helper 2 cells from SI and LI of untreated and TCA-treated mice were shown. (**G**) Representative images; (**H**) percentage and (**I**) absolute number of CD4+ IL17A+ T helper 17 cells from SI and LI of untreated and TCA-treated mice were shown. Data shown are combined from 2 independent experiments for a total of 7 to 10 mice per group. Data represent mean ± SEM. The statistical signficance of differences between groups was determined by the Mann-Whitney U test with * *p* ≤ 0.05, and *** *p* ≤ 0.001.

**TCA significantly decreased Th2 and Th17 cells in the intestine.** In addition to innate immunity, adaptive immune cells, mainly Th1 and Th17 cells, play an important role in controlling CA in the mucosal tissues [23,24,25,26,27,28]. To identify if TCA affects the number of T cells in the intestine, CD4 T helper cells were examined in the control and TCA-treated groups. No significant decrease in CD4+ IFNγ+ T helper 1 cells was noticed in the TCA treated mice (Figure 2A–C). The percentage and absolute number of CD4+ IL5+ T helper 2 cells were significantly decreased in the small but not in the large intestine of TCA-treated mice infected with CA (Figure 2D–F). The percentage and absolute number of CD4+ IL17+ T helper 17 cells were significantly decreased in the small and large intestine of TCA-treated mice in comparison to the untreated control groups (Figure 2G–I). Previous findings suggest that Th2 cells are not associated with protective immunity against CA [29]. On the other hand, Th17 cells play a critical role in controlling CA in the mucosal tissues [23,27,28]. Our findings reveal that TCA may control CA via inhibition of T helper 17 cells in the intestine.

Taken together, our findings suggest that TCA dysregulates intestinal phagocytes and Th17 cells that are important in the fight against CA. In the intestine, TCA is mainly absorbed and acts through the apical sodium-dependent bile acid transporter (ASBT) and farnesoid X receptor (FXR), respectively, expressed in small and large intestinal epithelial cells [30,31,32]. Furthermore, TCA and (or) FXR activation downregulates host immunity [33,34,35,36]. Findings from our lab suggest that TCA treatment increased the relative expression of genes encoding ASBT and FXR receptors in the colon of CA-infected mice [14]. In addition, antibiotic treatment considerably increases the expression of these receptors [31,37]. Based on these findings, we predict that TCA may induce CA colonization by dampening mucosal immune response through ASBT and FXR expressed in the intestine. Not only does antibiotic treatment increase TCA levels [13,14,38,39], but immunocompromised patients, such as individuals undergoing allogeneic hematopoietic cell transplantation (allo-HCT), who are highly susceptible to CA infection, have increased levels of TCA [4,40]. Therefore, future studies to understand (i) how TCA and bile acid receptors (ASBT and FXR) regulate host defense; and (ii) the effect of TCA on the regulation of other host immune factors such as regulatory T cells, B cells, dendritic cells, and innate lymphoid cells to control CA colonization in the intestine is critical to developing novel therapeutics to prevent and treat invasive CA infections in humans. Potential therapeutics include (i) modulating bile acid levels directly or through gut commensal bacteria such as *Lactobacillales* and *Bifidobacteriales* members that deconjugates TCA in the intestine [41,42,43] (ii) developing novel small molecule inhibitors against ASBT and FXR bile acid receptors, and (iii) using microbiota and immunomodulators to boost antifungal defenses such as CX3CR1+ mononuclear phagocytes to control CA in the intestine.

## Data Availability

Not applicable.

## Supplementary Materials

The following supporting information can be downloaded at: https://www.mdpi.com/article/10.3390/jof8060610/s1, File containing Supplementary Figure S1. Oral administration of TCA significantly increased the fungal load in feces.
