# Supplementary material for: Bile Acid Regulates Mononuclear Phagocytes and T Helper 17 Cells to Control Candida albicans in the Intestine
Source: J Fungi (Basel). 2022 Jun 7;8(6):610. doi: 10.3390/jof8060610 (PMC9224641; doi:10.3390/jof8060610)
Supplement: Supplementary file 1 [file jof-08-00610-s001.zip › jof-1756191-supplementary.pdf]

Supplementary Materials

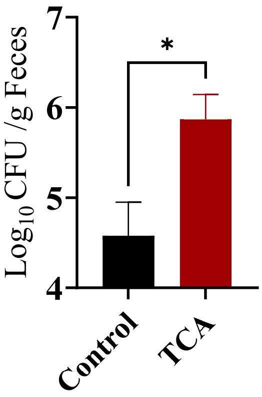

Supplementary Figure S1. Oral administration of TCA significantly increased the fungal load in feces. Groups of mice were infected with ~1 × 10^7 CFU of CA SC5314 via oral gavage. The control group received sterile drinking water and the TCA group received drinking water containing 1% TCA. Seven days post-infection and treatment, fecal pellets were collected and plated onto antibiotic-containing YPD plates to determine the fungal CFUs. Data represents mean ± SEM of 6-8 mice per group. * indicates a significant difference ($P \leq 0.05$) as determined by Mann-Whitney U test.
